# Supplementary material for: A cancer stem cell-like phenotype is associated with miR-10b expression in aggressive squamous cell carcinomas
Source: Cell Commun Signal. 2020 Apr 10;18:61. doi: 10.1186/s12964-020-00550-9 (PMC7146875; doi:10.1186/s12964-020-00550-9)
Supplement: Supplementary file 1 — Additional file 1. Supplementary information and data: Additional information on cell lines, bioinformatic data processing, CRISPR/Cas9-mediated knock-out of DIAPH2, flow cytometry for CD44 and CD24 expression, miRNA library preparation and next generation sequencing, Luciferase-reporter assay. Supplementary data summarized in tables: cell lines, antibodies, primers sequences, top up- and down-regulated miRNAs in microarray analysis: RDEB-cSCC versus RDEB-KCs, and HC-cSCC versus HC-KC, miR-10b target selection and validation. Supplementary experimental data in figures: miR-10b target selection and validation; controls for miRNA probe specificity in in situ hybridization on FFPE tissue sections; miRNA probe specificity for in situ hybridization on cultured cells and single cell resolution image-based data analysis workflow; miRNA probe specificity for in situ hybridization on cultured cells; generation of experimental cell lines; characterization of experimental cell lines; validation of miR-10b targets HOXD10 and DIAPH2; survival analysis and target evaluation. [file 12964_2020_550_MOESM1_ESM.docx]

***A cancer stem cell-like phenotype is associated with miR-10b expression in aggressive squamous cell carcinomas***

Monika Wimmer^1^* (mo.wimmer@salk.at), Roland Zauner^1^* (rolan.zauner@salk.at), Michael Ablinger^1^ (m.ablinger@salk.at), Josefina Piñón-Hofbauer^1^ (j.d.pinon@salk.at), Christina Guttmann-Gruber^1^ (c.gruber@salk.at), Manuela Reisenberger^1^ (m.reisenberger@salk.at), Thomas Lettner^1^ (t.lettner@salk.at), Norbert Niklas^2^ (norbert.niklas@o.roteskreuz.at), Johannes Proell^3^ (johannes.proell@jku.at), Mila Sajinovic^4^ (mila.sajinovic@unsw.edu.au), Paul De Souza^4,5^ (p.desouza@westernsydney.edu.au), Stefan Hainzl^1^ (s.hainzl@salk.at), Thomas Kocher^1^ (t.kocher@salk.at), Eva M. Murauer^1^ (e.murauer@salk.at), Johann W. Bauer^1,6^ (joh.bauer@salk.at), Dirk Strunk^7^ (dirk.strunk@pmu.ac.at), Julia Reichelt^1^ (julia.reichelt@newcastle.ac.uk), Albert S. Mellick^4,8^ (a.mellick@unsw.edu.au), Verena Wally^1#^ (v.wally@salk.at)

^1^Research Program for Molecular Therapy of Genodermatoses, EB House Austria, Department of Dermatology and Allergology, University Hospital of the Paracelsus Medical University, 5020 Salzburg, Austria.

^2^ Red Cross Transfusion Service of Upper Austria, 4020 Linz, Austria.

^3^ Center for Medical Research, Medical Faculty, Johannes-Kepler-University, 4020 Linz, Austria.

^4^ Medical Oncology, Ingham Institute for Applied Medical Research, Liverpool NSW 2170, Australia.

^5^ School of Medicine, Western Sydney University, Campbelltown, NSW 2560, Australia.

^6^Department of Dermatology and EB House Austria, University Hospital of the Paracelsus Medical University Salzburg, 5020 Salzburg, Austria.

^7^Institute of Experimental & Clinical Cell Therapy, Paracelsus Medical University, 5020 Salzburg, Austria.

^8^ School of Medicine, University of New South Wales, Kensington NSW 2052, Australia.

**MW and RZ equally contributed to the work.*

Supplementary online content

**Supplementary Information**

Cell lines

Bioinformatic data processing

CRISPR/Cas9-mediated knock-out of *DIAPH2*

Flow cytometry for CD44 and CD24 expression

miRNA library preparation and next generation sequencing

Luciferase-reporter assay

**Supplementary Tables**

Supplementary Table S1: Cell lines.

Supplementary Table S2: Antibodies.

Supplementary Table S3: Primers sequences.

Supplementary Table S4: Top up- and downregulated miRNAs in microarray analysis: RDEB-cSCC versus RDEB-KCs.

Supplementary Table S5: Top up- and downregulated miRNAs in microarray analysis: HC-cSCC versus HC-KCs.

Supplementary Table S6: MiR-10b target selection and validation.

**Supplementary Figures**

Supplementary Figure S1: MiR-10b target selection and validation.

Supplementary Figure S2: Controls for miRNA probe specificity in *in situ* hybridization on FFPE tissue sections.

Supplementary Figure S3: MiRNA probe specificity for *in situ* hybridization on cultured cells and single cell resolution image-based data analysis workflow.

Supplementary Figure S4: MiRNA probe specificity for *in situ* hybridization on cultured cells.

Supplementary Figure S5: Generation of experimental cell lines.

Supplementary Figure S6: Characterization of experimental cell lines.

Supplementary Figure S7: Validation of miR-10b targets HOXD10 and DIAPH2.

Supplementary Figure S8: Survival analysis and target evaluation.

**Supplementary references**

**Supplementary Information**

**Cell lines**

In this study we utilized cancer cells derived from primary skin tumors of RDEB, otherwise healthy, non-RDEB patients, as well as RDEB- and healthy control keratinocytes (HC-KCs). All RDEB cancer patients have in the meantime succumbed to the metastatic disease, underlining the devastating consequences of this genetic condition. Cell lines used for non-RDEB cSCCs have diverse characteristics. A431 (ATCC, CRL-1555) were derived from an epidermoid carcinoma from a 85 year old female patient and express high levels of epidermal growth factor receptor (EGFR) (1). When injecting A431 cells into mice, in one out of six mice a metastatic lesion was detected (2).

SCC13 were generated by Rheinwald *et al*. from a facial lesion of a female 56-year old female patient, who had received radiotherapy before excision (3). While we have no knowledge about the fate of the patient, SCC13 cells have been shown to be able to metastasize in a mouse xenograft model (4).

WT18SCC were isolated from a facial primary, moderately differentiated SCC of a female 66-year old patient. In the course of histology it was found that a BCC was associated at a tangential section plane. Because of that, it can not be excluded, that a minor proportion of BCC cells are present in the cell cultures. In a second surgery, histology also revealed presence of an actinic keratosis, which could not be removed in healthy complete. However, during the patient’s follow-up, lymph node metastasis occurred which were treated by complete dissection. The patient has now been tumor-free for about 3 years (Supplementary Table S1).

**Bioinformatic data processing**

MiRNome analysis and microarray raw data (.CEL files) were pre-processed using the robust multi-array average (RMA, oligo package, v1.46.0) background correction, normalization, log2-transformation and annotated with miRNA-4_1-st-v1 configuration file. Unsupervised clustering was performed using principal component analysis (PCA, FactoMineR v1.41) and factor loadings were used to identify miRNAs as drivers of experimental group separation in dimension 1. Differential expression of batch effect corrected (SVA package v3.30.0) miRNA with expression log2(signal) > 2.5 in at least 3 samples was assessed via Linear Models for Microarray Data (limma, v3.38.3). Overrepresentation analysis (clusterProfiler, v3.10.1) of miR-10a/b targetome (miRTarbase v.6.1) was performed on MSigDB hallmark gene sets (v6.1 Broad Institute, Massachusetts Institute of Technology) and significance (p ≤ 0.05; hypergeometric test) of enriched genesets were presented in a radial plot for either miR-10a, miR-10b or shared targets.

Data driven miR-10a/b target identification – transcriptome microarray raw data (.CEL files) were pre-processed by RMA (oligo package, v1.46.0) background correction, normalization, log2-transformation and annotated with Clariom_D_Human.na36.hg38.transcript file. Greater than 2-fold de-regulated mRNAs (limma) in RDEB-cSCC versus RDEB-KC were matched to union of miR-10a and miR-10b targets reported in TargetScan (v7.2) and miRTarbase (v6.1). The resulting predicted target list was prioritized by strength of inverse correlation (Pearson correlation) between mean of miR-10a/b (correlation of microarray log2 miRNA expression signals with TaqMan qPCR ΔCq values of respective miR-10 family members supports the preferential use of average miR-10 microarray signals, Supplementary Fig. S7E) and target mRNA log2-transformed, normalized expression signals. Final candidate selection was based on investigating the impact of miR-target gene expression on survival time of head and neck SCC (HNSCC) patients. HNSCC RNAseq and clinical data was retrieved from The Cancer Genome Atlas (TCGA, RTCGAToolbox, v2.12.1) and filtered for tumor stage IV and reports of lymphnode metastasis (N3 - TNM tumor staging). Patients were stratified by expression levels of prior short-listed miR-target genes into high and mid/low expression groups (75% quantile cut-off) and difference in survival time curves was tested applying log-rank test (survival, 2.43-3).

For scientific literature driven miR-10b target identification, the search term “(cancer) AND (miR-10b)” was used to screen (RISmed, v2.1.7) PubMed articles. Biological terms were extracted from abstract text bodies through named entity recognition by accessing geniatagger (v3.0, School of Computer Science, University of Manchester). Terms were summarized and highlighted by frequency of their occurance in abstracts (wordcloud, v0.2.1).

For *DIAPH2* expression analysis in RDEB-cSCC and RDEB-skin tissue, normalized RNAseq data generated by Prof. Andy South’s lab (Thomas Jefferson University, USA) was retrieved from GEO repository (GEOquery, v2.50.5, GSE111582) and filtered for *DIAPH1,2,3* and *HOXD10* log2-transformed expression signals.

Data evaluation and analysis of miRNA-seq data was performed via R package DESeq2. Therefore read counts were loaded from .csv files into the statistical program language R (v 3.3.1) for all further processing and data analysis steps. Multiple annotations deriving from 2 bp mismatches were resolved for final read count summary. Annotation codes were adapted for further database screenings, like adding the prefix "hsa-". Normalization and inference testing with fold change estimation was performed via R package "DEseq2" (v 1.12.3). Results were reported in box and dotplots created by "ggplot2" (v 2.1.0).

**CRISPR/Cas9-mediated knock-out of DIAPH2**

DIAPH2 knock-out cell lines were generated using the U6gRNA-Cas9-2A-RFP vector (Sigma Aldrich, HS000336959), including a guide RNA specifically targeting *DIAPH2* exon 1 (Supplementary Table S3). Upon Xfect-mediated transfection of 1090KC, efficiency in the bulk was analyzed using a T7-endonuclease I (New England Biolabs, E3321), according to the manufacturer’s instruction and as describe elsewhere (5). Primers were designed to flank the target region, and which will produce two fragments of different sizes upon T7-endonuclease I digest (Supplementary Table S3). Clones were generated by minimal dilution and knock-out was confirmed by Sanger sequencing and Western blot analysis and semi-quantitative (sq)RT-PCR, as described.

**Flow cytometry for CD44 and CD24 expression**

10^6^ cells were re-suspended in PBS and double stained with CD44-FITC (FITC anti-mouse/human CD44, clone:IM7, Cat.103005, Lot.B228503) and CD24-PE (PE anti human CD24, clone:ML5, BioLegend, Cat.311105, Lot.B216880). Cytometry was performed on a Gallios Flow Cytometer (Beckman Coulter) and data were analysed using the Kaluza analysis software (Beckman Coulter). Isotype controls were included to exclude non-specific staining (PE Mouse IgG2a, k isotype ctr., clone:MOPC-173, Cat.400211, Lot.B265821; FITC Rat IgG2b, k isotype ctr., clone:RTK4530, Cat.400605, Lot.B265825).

**miRNA library preparation and next generation sequencing**

*miRNA library preparation*

cDNA libraries from ~50 ng AGO2 co-immunoprecipitated small RNA pools were generated using the NEBNext Multiplex Small RNA Library Prep Set for Illumina (Set2; NEB #E7580S/L) according to the manufacturer’s protocol, including quality control and polyacrylamide gel-based size selection steps. The cDNA libraries were purified using the “QIAquick PCR purification kit” (Qiagen, Cat No./ID: 2810). Concentrations of miRNA and cDNA were analyzed using a Qubit fluorometer and quality controls of cDNA libraries were done using an Experion™ Automated electrophoresis system (Bio-Rad) using the “Experion DNA 1K analysis kit” (Experion, #700-7107) following the manufacturer’ instruction manual.

*miRNA next generation sequencing*

All libraries were subjected to Illumina next-generation sequencing (NGS) on an Illumina MiSeq platform. For the generation of the final sequencing libraries, up to four samples were simultaneously equimolarly pooled. Molarities for pooling were calculated by fragment size (Agilent) and quantification using PicoGreen (Thermo Fisher, P11496). The final concentration of the sequencing libraries were 12 pM in which 1% depicted the spike-in of PhiX sequencing control. Sequencing itself was performed according to the manufacturer’ instruction by using a 50-cycle v2 MiSeq kit (Illumina, MS-102-3001). The sequencing pipeline included base calling and demultiplexing, generating a total of 92.2 M reads (in six runs) with Q30 ranging from 90.6 % to 97.1 % and an average error rate of 0.33 % (measured by PhiX control). Adapters contained in the sample sequences were trimmed after sequencing, using the CLC Genomics Workbench 7 (Qiagen). After trimming, the size distribution showed a typical miRNA pattern (around 22-25 bp), no additional filters were applied. The trimmed sequences were counted and grouped (by equal bp). Annotated sequences were identified by miRBase 21 and reported according to the database annotation. Mature length variants were determined by using the default settings of the "Annotate and Merge Counts" (2 bp tolerance). Reports included miRNA name and maturity as well as their abundance. Unmatched sequences (without annotation) were mapped against the whole human genome (using default parameters). Results were provided in excel and comma-separated-value (.csv) files, including all sequences of identified RNAs and the respective annotation if available.

**Luciferase-reporter assay**

For Dual-Luciferase® Reporter (DLR™) assay system (Promega, E1910) 3’UTRs of *DIAPH2* and *HOXD10*, respectively, were cloned into the pmirGLO Dual-Luciferase miRNA Target Expression Vector (Promega), downstream the firefly luciferase coding region. For primers see Supplementary Table S3. 5 µg of reporter vector were co-transfected with 100 pmol miR-10b-5p mimic, or a scrambled control (SCR) into primary HC-KCs at ~70 % confluency in a 6-well format. Cells were lysed in passive lysis buffer after 24 hrs and luminescence was measured using the Tecan Spark 10M plate reader. Luciferase signals were normalized to Renilla signals for comparison of co-transfection with miR-10b or SCR.

**Supplementary Tables**

**Supplementary Table S1: Cell lines.**

|  | **age (yrs)** | **sex** | ***COL7A1* mutation**  **(allele 1 / allele 2)** | **localization** | **tumor type / history of metastasis / metastatic potential** | **reference** |
| --- | --- | --- | --- | --- | --- | --- |
| **1090KC** | 23 | m | healthy control | arm | - | - |
| **SKC013** | 35 | f | healthy control | abdominal | - | - |
| **SKC015** | 36 | f | healthy control | abdominal | - | - |
| **SKC018** | 31 | m | healthy control | abdominal | - | - |
| **1102KC** | 38 | f | healthy control | lower leg | - | (6),  (NHK 3) |
| **RDEB-01KC** | 20 | f | c.2005C>T; p.R669X | leg | - | - |
| **RDEB-03KC** | 18 | m | c.425A>G / c.5440G>T  p.K142R / p.R1814C | right inner upper arm | - | - |
| **RDEB-29KC** | 2 | m | c.425A>G / c.520G>A  p.L142R / p.G174R | foreskin | - | - |
| **RDEB-30KC** | 2 | m | c.425A>G / c.520G>A  p.L142R / p.G174R | foreskin | - | - |
| **RDEB-43KC** | 17 | f | c.4027C>T / c.425A>G  p.R1343X / p.K142R | right inner thigh | - | - |
| **RDEB-53KC** | 32 | f | c.2005C>T, homozygous | unknown | - | - |
| **RDEB-55KC** | 21 | m | c.976+4A>C  p.INS34AA | left upper thigh | - | - |
| **RDEB-57KC** | 6 | w | c.427-2A>G / c.4172insC;  p. splice site / p.1391fsX10 | unknown | - | - |
| **RDEB-SCC1** | 32 | f | c.8244dupC  homozygous | SCC shoulder | unknown | (7), (RDEB6) |
| **RDEB-SCC2** | 54 | m | c.3832-1 G>A /  undetermined | SCC hand | primary,  poorly differentiated,  development of metastatic disease | (7), (RDEB3) |
| **RDEB-SCC53** | 31 | f | c.2005C>T  homozygous | SCC elbow | primary,  development of metastatic disease | - |
| **RDEB-SCC62** | 29 | f | c.682+1G>A / c.7474C>T  p.R2492X | SCC lower arm | primary, highly differentiated,  development of metastatic disease | (5) |
| **A431** | 85 | f | otherwise healthy | cSCC | primary,  metastasize in mouse model | ATCC |
| **SCC13** | 56 | f | otherwise healthy | cSCC facial | radiation before removal,  metastasize in mouse model | (3) |
| **WT18SCC** | 66 | f | otherwise healthy | cSCC facial with tangential BCC | primary,  moderatly differentiated,  lymph node metastasis | - |
| **DDEB-22KC** | 46 | m | p.G2043R | right inner upper arm | - | - |
| **DDEB-25KC** | 25 | m | c. 6127G>A/ homozygous  p.G2042R | left inner upper arm | - | - |

Several cell lines from different donors were used, including RDEB-cSCC (n = 4), HC-cSCC (n = 3), RDEB-KC (n = 8), DDEB-KC (n = 2) and HC-KCs (n = 5) lines. Patients’ age, gender, and - where applicable - tumor stadium, history of metastasis of metastatic potential in a mouse model, as well as tumor localization are given. For RDEB-patients, respective *COL7A1* mutations are included.

**Supplementary Table S2: Antibodies.**

| **antigen / host species** | **catalog number / Lot** | **clone no** | **company** | **reference** |
| --- | --- | --- | --- | --- |
| **Antibodies for Western blot analysis** | | | | |
| **primary antibodies** | | | | |
| DIAPH2 / rabbit | HPA005647  Lot A37866 | polyclonal | Sigma Life Sciences | (8) |
| TWIST1 / rabbit | T6451  Lot 035M4793V | polyclonal | Sigma Life Sciences | (15) |
| Histone 3 / rabbit | PA5-16183  Lot SB2344305B | polyclonal | Thermo Fisher Scientific | (9) |
| Annexin 1 / mouse | sc-12740  Lot J1216 | EH17a | Santa Cruz | (10) |
| **secondary antibodies** | | | | |
| HRP-labelled  goat-α-rabbit IgG2b | K4010  Lot 10072150 |  | Dako Envision |  |
| HRP-labelled  goat-α-mouse IgG2b | K4004  Lot 10076454 |  | Dako Envision |  |
| **Antibodies for immunofluorescence microscopy** | | | | |
| **primary antibodies** | | | | |
| DIAPH2 / mouse | sc-55539  Lot B0717 | B-11 | Santa Cruz | (11) |
| HOXD10 / rabbit | sc-66926  Lot A0510 | polyclonal | Santa Cruz | (12) |
| CD31 / rabbit | PA5-16301  Lot NJ1608523 | polyclonal | Thermo Fisher Scientific | (13) |
| CD45 / mouse | MAB1430  Lot ILP071808A | 2D1 | R&D Systems | (14) |
| TWIST1 / rabbit | T6451  Lot 035M4793V | polyclonal | Sigma Life Sciences | (15) |
| Pan-KRT 14,15,16,19 (A647) / mouse | 563648  Lot 8003894 | KA4 | BD Pharmingen | (16) |
| **secondary antibodies** | | | | |
| Alexa Fluor®488  goat-α-mouse IgG (H+L) | A11001  Lot 1939600 | polyclonal | Thermo Fisher Scientific | (17) |
| Alexa Fluor®594  goat-α-rabbit IgG (H+L) | A11037  Lot 1608397 | polyclonal | Thermo Fisher Scientific | (18) |
| Alexa Fluor®488  goat-α-rabbit IgG (H+L) | A11008  Lot 1829920 | polyclonal | Thermo Fisher Scientific | (19) |

**Supplementary Table S3: Primer sequences.**

| **Primers for sqRT-PCR** | |
| --- | --- |
| GAPDH_forward | 5’ GCCAACGTGTCAGTGGTGGA 3’ |
| GAPDH_reverse | 5’ CACCACCCTGTTGCTGTAGCC 3’ |
| TUBA1_forward | 5’ATGGAGCCCTGAATGTTGAC3’ |
| TUBA1_reverse | 5’ CTCAAAGCAAGCATTGGTGA 3’ |
| DIAPH2_forward | 5’ GCAGATGATGTGCGTGACCG 3’ |
| DIAPH2_reverse | 5’ GTTAAGGTTCATGTCCTCCATCATTTTTTCAAAGAG 3’ |
| HOXD10_forward | 5’ CCGCAGCTCTCCGCT 3’ |
| HOXD10_reverse | 5’ TCAGACTTGATTTCCTCTTTGCTTTCCTTC 3’ |
| **Primers for cloning** | |
| HOXD10 3’UTR_forward | 5’ GAGCTCGTCTGAGGCCGGT 3’ |
| HOXD10 3’UTR_reverse | 5’ GTCGACGAACTCATTTCCAGAG 3’ |
| DIAPH2 3’UTR forward | 5’ GAGCTCTTCCTGATGCCAAAG 3’ |
| DIAPH2 3’UTR reverse | 5’ GTCGACTAACTTTTACTCCCTA 3’ |
| miR10b_EcoRI_fw | 5’ GATCGAATTCCCGAGGTTGTAACGTTGTC 3’ |
| miR10b_XhoI_rv | 5’ GATCCTCGAGCAAAAATGAAGTTTTTG 3’ |
| **Primers for T7 endonuclease I assay** | |
| DIAPH2genEx1_fw | 5’ CTAGTTCCCTCCCTGTTCTCGCTG 3’ |
| DIAPH2genEx1_rv | 5’ CGTGTTCAATGGCAGGAGGATCCC 3’ |
| **gRNAs for CRISPR** | |
| gRNA1_MIR10B_fw | 5´-TAATACGACTCACTATAGtgaggtacctaggtcgctgc-3´ |
| gRNA1_MIR10B_rv | 5´-TTCTAGCTCTAAAACGCAGCGACCTAGGTACCTCA-3´ |
| gRNA2_MIR10B_fw | 5´-TAATACGACTCACTATAGCTAGTCTCCATGTCGCACTT-3´ |
| gRNA2_MIR10B_rv | 5´-TTCTAGCTCTAAAACAAGTGCGACATGGAGACTAG-3´ |
| gRNA_DIAPH2 | 5’-GCAGCGAGGAACCCGGTGG-3’ |

**Supplementary Table S4: Top up- and downregulated miRNAs in microarray data analysis.**

**RDEB-cSCC (n=4) versus RDEB-KCs (n=6)**

| **miRNA** | **log2(FC)** | **<log2(Signal)>** | **p-value** | **FDR** | **miRBase Sequence** |
| --- | --- | --- | --- | --- | --- |
| hsa-miR-10a-5p | 3.15 | 2.75 | 3.09e-07 | 1.57e-04 | UACCCUGUAGAUCCGAAUUUGUG |
| hsa-miR-146a-5p | 2.99 | 2.32 | 4.86e-03 | 5.66e-02 | UGAGAACUGAAUUCCAUGGGUU |
| hsa-miR-193a-3p | 2.04 | 2.51 | 1.30e-03 | 2.89e-02 | AACUGGCCUACAAAGUCCCAGU |
| hsa-miR-4485 | 1.99 | 5.23 | 7.75e-03 | 7.07e-02 | UAACGGCCGCGGUACCCUAA |
| hsa-miR-194-5p | 1.74 | 4.04 | 3.20e-04 | 1.03e-02 | UGUAACAGCAACUCCAUGUGGA |
| hsa-miR-941 | 1.71 | 3.04 | 2.52e-04 | 1.03e-02 | CACCCGGCUGUGUGCACAUGUGC |
| hsa-miR-3929 | 1.70 | 2.06 | 1.40e-02 | 1.02e-01 | GAGGCUGAUGUGAGUAGACCACU |
| hsa-let-7d-3p | 1.68 | 3.04 | 2.33e-04 | 1.03e-02 | CUAUACGACCUGCUGCCUUUCU |
| hsa-miR-421 | 1.50 | 4.60 | 2.49e-03 | 4.16e-02 | AUCAACAGACAUUAAUUGGGCGC |
| hsa-miR-34c-5p | 1.48 | 2.92 | 1.73e-02 | 1.18e-01 | AGGCAGUGUAGUUAGCUGAUUGC |
| hsa-miR-1973 | 1.45 | 2.59 | 3.23e-03 | 4.58e-02 | ACCGUGCAAAGGUAGCAUA |
| hsa-miR-324-5p | 1.42 | 5.90 | 1.75e-03 | 3.23e-02 | CGCAUCCCCUAGGGCAUUGGUGU |
| hsa-miR-491-5p | 1.42 | 2.52 | 1.63e-03 | 3.23e-02 | AGUGGGGAACCCUUCCAUGAGG |
| hsa-miR-15a-5p | 1.40 | 4.86 | 7.23e-03 | 6.92e-02 | UAGCAGCACAUAAUGGUUUGUG |
| hsa-miR-99a-5p | 1.39 | 4.07 | 1.70e-02 | 1.18e-01 | AACCCGUAGAUCCGAUCUUGUG |
| hsa-miR-619-5p | 1.38 | 4.15 | 6.98e-03 | 6.84e-02 | GCUGGGAUUACAGGCAUGAGCC |
| hsa-miR-139-5p | 1.36 | 2.29 | 3.38e-02 | 1.69e-01 | UCUACAGUGCACGUGUCUCCAGU |
| hsa-miR-23b-5p | 1.34 | 3.01 | 2.62e-02 | 1.47e-01 | UGGGUUCCUGGCAUGCUGAUUU |
| hsa-miR-34c-3p | 1.31 | 3.37 | 4.28e-02 | 1.93e-01 | AAUCACUAACCACACGGCCAGG |
| hsa-miR-132-3p | 1.26 | 5.74 | 7.01e-03 | 6.84e-02 | UAACAGUCUACAGCCAUGGUCG |
| hsa-miR-212-3p | 1.26 | 1.96 | 3.34e-03 | 4.58e-02 | UAACAGUCUCCAGUCACGGCC |
| hsa-miR-769-5p | 1.23 | 2.28 | 1.35e-03 | 2.89e-02 | UGAGACCUCUGGGUUCUGAGCU |
| hsa-miR-501-5p | 1.18 | 3.03 | 3.46e-03 | 4.64e-02 | AAUCCUUUGUCCCUGGGUGAGA |
| hsa-miR-188-5p | 1.18 | 3.08 | 1.42e-02 | 1.02e-01 | CAUCCCUUGCAUGGUGGAGGG |
| hsa-miR-425-3p | 1.16 | 3.48 | 4.56e-03 | 5.56e-02 | AUCGGGAAUGUCGUGUCCGCCC |
| ***hsa-miR-10b-5p*** | ***1.15*** | ***2.31*** | ***2.24e-02*** | ***1.37e-01*** | ***UACCCUGUAGAACCGAAUUUGUG*** |
| hsa-miR-140-3p | 1.15 | 5.02 | 1.02e-02 | 8.54e-02 | UACCACAGGGUAGAACCACGG |
| hsa-miR-342-5p | 1.12 | 2.39 | 1.01e-02 | 8.54e-02 | AGGGGUGCUAUCUGUGAUUGA |
| hsa-miR-192-5p | 1.12 | 2.80 | 3.47e-04 | 1.03e-02 | CUGACCUAUGAAUUGACAGCC |
| hsa-miR-4749-5p | 1.10 | 4.33 | 2.37e-02 | 1.40e-01 | UGCGGGGACAGGCCAGGGCAUC |
| hsa-miR-6124 | 1.07 | 4.32 | 1.28e-02 | 9.68e-02 | GGGAAAAGGAAGGGGGAGGA |
| hsa-miR-151b | 1.05 | 6.32 | 5.12e-05 | 4.09e-03 | UCGAGGAGCUCACAGUCU |
| hsa-miR-671-3p | 1.04 | 3.35 | 1.33e-02 | 9.87e-02 | UCCGGUUCUCAGGGCUCCACC |
| hsa-miR-877-5p | 1.03 | 3.87 | 4.11e-02 | 1.88e-01 | GUAGAGGAGAUGGCGCAGGG |
| hsa-miR-3613-3p | -1.00 | 7.44 | 1.87e-04 | 1.00e-02 | ACAAAAAAAAAAGCCCAACCCUUC |
| hsa-miR-4532 | -1.05 | 6.12 | 2.67e-02 | 1.47e-01 | CCCCGGGGAGCCCGGCG |
| hsa-miR-4674 | -1.07 | 6.43 | 6.23e-03 | 6.55e-02 | CUGGGCUCGGGACGCGCGGCU |
| hsa-miR-205-5p | -1.10 | 12.07 | 8.65e-03 | 7.60e-02 | UCCUUCAUUCCACCGGAGUCUG |
| hsa-miR-200b-3p | -1.16 | 5.09 | 3.77e-02 | 1.79e-01 | UAAUACUGCCUGGUAAUGAUGA |
| hsa-miR-99b-3p | -1.23 | 4.38 | 2.25e-02 | 1.37e-01 | CAAGCUCGUGUCUGUGGGUCCG |
| hsa-miR-296-3p | -1.27 | 3.26 | 2.37e-02 | 1.40e-01 | GAGGGUUGGGUGGAGGCUCUCC |
| hsa-miR-1298-3p | -1.27 | 2.61 | 6.04e-03 | 6.47e-02 | CAUCUGGGCAACUGACUGAAC |
| hsa-let-7e-5p | -1.30 | 9.30 | 8.09e-03 | 7.22e-02 | UGAGGUAGGAGGUUGUAUAGUU |
| hsa-miR-193b-5p | -1.35 | 5.55 | 7.18e-04 | 1.83e-02 | CGGGGUUUUGAGGGCGAGAUGA |
| hsa-miR-424-3p | -1.36 | 7.80 | 2.77e-03 | 4.24e-02 | CAAAACGUGAGGCGCUGCUAU |
| hsa-miR-200a-5p | -1.54 | 4.00 | 4.13e-03 | 5.28e-02 | CAUCUUACCGGACAGUGCUGGA |
| hsa-miR-125a-5p | -1.61 | 9.14 | 1.11e-02 | 8.79e-02 | UCCCUGAGACCCUUUAACCUGUGA |
| hsa-miR-200b-5p | -1.70 | 5.03 | 1.45e-03 | 2.99e-02 | CAUCUUACUGGGCAGCAUUGGA |
| hsa-miR-99b-5p | -1.94 | 8.49 | 2.33e-02 | 1.40e-01 | CACCCGUAGAACCGACCUUGCG |
| hsa-miR-34a-5p | -2.26 | 5.51 | 6.93e-03 | 6.84e-02 | UGGCAGUGUCUUAGCUGGUUGU |

**Supplementary Table S5: Top up- and downregulated miRNAs in microarray data analysis.**

**HC-cSCC (n=3) versus HC-KCs (n=4)**

| **miRNA** | **log2(FC)** | **<log2(Signal)>** | **p-value** | **FDR** | **miRBase Sequence** |
| --- | --- | --- | --- | --- | --- |
| hsa-miR-139-5p | 2.72 | 2.29 | 6.87e-04 | 2.05e-02 | UCUACAGUGCACGUGUCUCCAGU |
| hsa-miR-4485 | 2.55 | 5.23 | 3.16e-03 | 4.13e-02 | UAACGGCCGCGGUACCCUAA |
| hsa-miR-877-5p | 2.28 | 3.87 | 3.39e-04 | 1.40e-02 | GUAGAGGAGAUGGCGCAGGG |
| hsa-miR-126-3p | 2.06 | 3.31 | 9.37e-03 | 6.79e-02 | UCGUACCGUGAGUAAUAAUGCG |
| hsa-miR-1285-3p | 1.99 | 1.99 | 2.94e-07 | 3.94e-05 | UCUGGGCAACAAAGUGAGACCU |
| hsa-miR-27b-5p | 1.97 | 3.32 | 2.02e-03 | 3.63e-02 | AGAGCUUAGCUGAUUGGUGAAC |
| hsa-miR-92b-5p | 1.95 | 4.26 | 7.85e-04 | 2.22e-02 | AGGGACGGGACGCGGUGCAGUG |
| hsa-miR-629-5p | 1.89 | 4.45 | 6.11e-03 | 6.06e-02 | UGGGUUUACGUUGGGAGAACU |
| hsa-miR-3615 | 1.88 | 2.16 | 1.34e-03 | 2.88e-02 | UCUCUCGGCUCCUCGCGGCUC |
| hsa-miR-941 | 1.86 | 3.04 | 3.85e-04 | 1.48e-02 | CACCCGGCUGUGUGCACAUGUGC |
| hsa-miR-491-5p | 1.77 | 2.52 | 6.75e-04 | 2.05e-02 | AGUGGGGAACCCUUCCAUGAGG |
| hsa-miR-21-5p | 1.76 | 4.44 | 2.66e-02 | 1.30e-01 | UAGCUUAUCAGACUGAUGUUGA |
| hsa-miR-140-3p | 1.76 | 5.02 | 9.78e-04 | 2.52e-02 | UACCACAGGGUAGAACCACGG |
| hsa-miR-629-3p | 1.69 | 2.86 | 4.67e-04 | 1.57e-02 | GUUCUCCCAACGUAAGCCCAGC |
| hsa-miR-151b | 1.69 | 6.32 | 4.26e-07 | 4.56e-05 | UCGAGGAGCUCACAGUCU |
| hsa-miR-193a-3p | 1.61 | 2.51 | 1.71e-02 | 9.98e-02 | AACUGGCCUACAAAGUCCCAGU |
| hsa-miR-484 | 1.60 | 2.39 | 2.43e-03 | 3.81e-02 | UCAGGCUCAGUCCCCUCCCGAU |
| hsa-miR-192-5p | 1.56 | 2.80 | 3.41e-05 | 2.28e-03 | CUGACCUAUGAAUUGACAGCC |
| hsa-miR-21-3p | 1.51 | 4.97 | 7.93e-03 | 6.26e-02 | CAACACCAGUCGAUGGGCUGU |
| hsa-miR-92b-3p | 1.49 | 7.08 | 2.81e-07 | 3.94e-05 | UAUUGCACUCGUCCCGGCCUCC |
| hsa-miR-146b-5p | 1.42 | 2.05 | 1.50e-02 | 9.32e-02 | UGAGAACUGAAUUCCAUAGGCU |
| hsa-miR-625-5p | 1.39 | 4.24 | 2.45e-02 | 1.23e-01 | AGGGGGAAAGUUCUAUAGUCC |
| hsa-miR-505-5p | 1.37 | 2.72 | 7.10e-03 | 6.24e-02 | GGGAGCCAGGAAGUAUUGAUGU |
| hsa-miR-10a-5p | 1.36 | 2.75 | 1.09e-02 | 7.40e-02 | UACCCUGUAGAUCCGAAUUUGUG |
| hsa-miR-3135b | 1.34 | 4.28 | 4.51e-02 | 1.83e-01 | GGCUGGAGCGAGUGCAGUGGUG |
| hsa-miR-2110 | 1.34 | 3.20 | 2.94e-02 | 1.36e-01 | UUGGGGAAACGGCCGCUGAGUG |
| hsa-miR-342-5p | 1.32 | 2.39 | 7.79e-03 | 6.26e-02 | AGGGGUGCUAUCUGUGAUUGA |
| hsa-miR-1307-3p | 1.29 | 7.08 | 2.41e-05 | 1.85e-03 | ACUCGGCGUGGCGUCGGUCGUG |
| hsa-miR-128-3p | 1.29 | 3.65 | 2.03e-02 | 1.12e-01 | UCACAGUGAACCGGUCUCUUU |
| hsa-miR-671-3p | 1.29 | 3.35 | 7.58e-03 | 6.26e-02 | UCCGGUUCUCAGGGCUCCACC |
| hsa-miR-151a-3p | 1.23 | 7.58 | 1.21e-07 | 3.23e-05 | CUAGACUGAAGCUCCUUGAGG |
| hsa-miR-148b-3p | 1.22 | 2.00 | 1.21e-03 | 2.83e-02 | UCAGUGCAUCACAGAACUUUGU |
| hsa-miR-151a-5p | 1.19 | 9.27 | 7.88e-09 | 4.22e-06 | UCGAGGAGCUCACAGUCUAGU |
| hsa-miR-330-3p | 1.18 | 5.02 | 3.20e-02 | 1.44e-01 | GCAAAGCACACGGCCUGCAGAGA |
| ***hsa-miR-10b-5p*** | ***1.16*** | ***2.31*** | ***3.97e-02*** | ***1.69e-01*** | ***UACCCUGUAGAACCGAAUUUGUG*** |
| hsa-miR-3682-3p | 1.14 | 2.04 | 4.80e-03 | 5.25e-02 | UGAUGAUACAGGUGGAGGUAG |
| hsa-miR-425-3p | 1.08 | 3.48 | 1.60e-02 | 9.53e-02 | AUCGGGAAUGUCGUGUCCGCCC |
| hsa-miR-421 | 1.06 | 4.60 | 4.41e-02 | 1.81e-01 | AUCAACAGACAUUAAUUGGGCGC |
| hsa-miR-135b-3p | 1.00 | 2.17 | 1.95e-02 | 1.11e-01 | AUGUAGGGCUAAAAGCCAUGGG |
| hsa-miR-4758-5p | -1.00 | 4.81 | 3.71e-03 | 4.42e-02 | GUGAGUGGGAGCCGGUGGGGCUG |
| hsa-miR-4497 | -1.01 | 10.04 | 6.71e-03 | 6.24e-02 | CUCCGGGACGGCUGGGC |
| hsa-miR-409-3p | -1.05 | 2.12 | 3.74e-02 | 1.61e-01 | GAAUGUUGCUCGGUGAACCCCU |
| hsa-miR-1343-5p | -1.10 | 5.00 | 7.95e-03 | 6.26e-02 | UGGGGAGCGGCCCCCGGGUGGG |
| hsa-miR-548a-3p | -1.11 | 2.80 | 1.67e-02 | 9.83e-02 | CAAAACUGGCAAUUACUUUUGC |
| hsa-miR-193b-3p | -1.14 | 8.74 | 3.26e-06 | 2.91e-04 | AACUGGCCCUCAAAGUCCCGCU |
| hsa-miR-3621 | -1.18 | 5.41 | 8.34e-03 | 6.39e-02 | CGCGGGUCGGGGUCUGCAGG |
| hsa-miR-6126 | -1.36 | 5.86 | 7.60e-03 | 6.26e-02 | GUGAAGGCCCGGCGGAGA |
| hsa-miR-6068 | -1.39 | 3.89 | 2.66e-03 | 3.85e-02 | CCUGCGAGUCUCCGGCGGUGG |
| hsa-miR-503-5p | -1.60 | 7.55 | 4.67e-05 | 2.78e-03 | UAGCAGCGGGAACAGUUCUGCAG |
| hsa-miR-4532 | -1.63 | 6.12 | 3.60e-03 | 4.39e-02 | CCCCGGGGAGCCCGGCG |
| hsa-miR-181b-5p | -1.72 | 8.06 | 2.36e-04 | 1.15e-02 | AACAUUCAUUGCUGUCGGUGGGU |
| hsa-miR-181a-5p | -1.88 | 8.19 | 2.68e-04 | 1.19e-02 | AACAUUCAACGCUGUCGGUGAGU |
| hsa-miR-424-3p | -1.91 | 7.80 | 4.16e-04 | 1.49e-02 | CAAAACGUGAGGCGCUGCUAU |
| hsa-miR-503-3p | -2.04 | 3.11 | 1.40e-03 | 2.88e-02 | GGGGUAUUGUUUCCGCUGCCAGG |
| hsa-miR-203a | -2.49 | 6.53 | 5.26e-03 | 5.64e-02 | GUGAAAUGUUUAGGACCACUAG |
| hsa-miR-708-5p | -3.50 | 7.93 | 9.89e-04 | 2.52e-02 | AAGGAGCUUACAAUCUAGCUGGG |

Microarray data was RMA background corrected & normalized, SVA batch corrected, foldchange, p-value and FDR were determined by limma package in R.

**Supplementary Table S6: miR-10b target selection and validation.**

| **gene** | **mRNA** | **P**  **(KM)** | **log2**  **(FC)** | **p-value** | **r (miR-10)** | **miR**  **Tarbase miR-10b** | **Target**  **Scan miR-10** |
| --- | --- | --- | --- | --- | --- | --- | --- |
| ***DIAPH2*** | **diaphanous-related formin 2 (DIAPH2), transcript variant 156** | **0.02** | **-3.43** | **8.30e-04** | **-0.57** | **TRUE** | **FALSE** |
| *RNF180* | ring finger protein 180 (RNF180), transcript variant 1 | 0.12 | -2.12 | 2.60e-07 | -0.72 | FALSE | TRUE |
| *EPN3* | epsin 3 (EPN3) | 0.22 | -1.54 | 1.39e-03 | -0.70 | FALSE | TRUE |
| *ZNF43* | zinc finger protein 43 (ZNF43), transcript variant 1 | 0.26 | -1.86 | 6.50e-04 | -0.77 | FALSE | TRUE |
| *RBPMS* | RNA binding protein with multiple splicing (RBPMS), transcript variant 1 | 0.34 | -2.46 | 2.60e-05 | -0.78 | FALSE | TRUE |
| *NT5DC3* | 5-nucleotidase domain containing 3 (NT5DC3) | 0.34 | -1.02 | 9.80e-05 | -0.81 | FALSE | TRUE |
| *ZNF470* | zinc finger protein 470 (ZNF470) | 0.38 | -1.13 | 2.10e-06 | -0.78 | FALSE | TRUE |
| *ZFP82* | ZFP82 zinc finger protein (ZFP82) | 0.51 | -1.07 | 1.65e-03 | -0.72 | FALSE | TRUE |
| *RORA* | RAR-related orphan receptor A (RORA), transcript variant 3 | 0.52 | -1.17 | 3.52e-03 | -0.69 | TRUE | TRUE |
| *ARHGEF9* | Cdc42 guanine nucleotide exchange factor (GEF) 9 (ARHGEF9), transcript variant 2 | 0.56 | -1.17 | 8.24e-03 | -0.64 | FALSE | TRUE |
| *SDC1* | syndecan 1 (SDC1), transcript variant 1 | 0.70 | -1.27 | 3.90e-06 | -0.70 | TRUE | TRUE |
| *GJA5* | gap junction protein, alpha 5, 40kDa (GJA5), transcript variant A | 0.73 | -1.30 | 4.07e-03 | -0.67 | FALSE | TRUE |
| *SPARC* | secreted protein, acidic, cysteine-rich (osteonectin) (SPARC), transcript variant 2 | 0.81 | -3.00 | 7.64e-03 | -0.67 | FALSE | FALSE |
| *SH3BGRL* | SH3 domain binding glutamate-rich protein like (SH3BGRL) | 0.85 | -2.33 | 1.30e-06 | -0.68 | FALSE | TRUE |
| *MAP9* | microtubule-associated protein 9 (MAP9) | 0.87 | -2.56 | 7.30e-04 | -0.82 | FALSE | TRUE |
| *SDK2* | sidekick cell adhesion molecule 2 (SDK2) | 0.93 | -1.19 | 4.00e-06 | -0.88 | FALSE | TRUE |
| *ALDH4A1* | aldehyde dehydrogenase 4 family, member A1 (ALDH4A1), transcript variant 3 | 0.95 | -1.02 | 5.10e-06 | -0.76 | FALSE | TRUE |
| *ADAMTS1* | ADAM metallopeptidase with thrombospondin type 1 motif, 1 (ADAMTS1) | 0.96 | -2.98 | 6.10e-05 | -0.74 | FALSE | TRUE |
| *RAB3B* | RAB3B, member RAS oncogene family (RAB3B) | 0.97 | -2.40 | 9.00e-05 | -0.83 | FALSE | TRUE |
| *KAZN* | kazrin, periplakin interacting protein (KAZN), transcript variant D | NA | -1.11 | 8.10e-06 | -0.78 | FALSE | TRUE |

Top 20 *in silico* predicted miR-10 targets, ranked by level of significance (log rank Kaplan-Meier p values, column “p(KM)”) of differences in survival curves when comparing high and mid/low TCGA RNA-seq gene expression datasets of metastatic HNSCC samples. Columns “log2(FC)” and “p-value” indicate foldchange and significance between gene expression of RDEB-cSCC and RDEB-KC based on the microarray transcriptome data. Pearson correlation (r) between mean of miR-10a/b log2-transformed normalized microarray expression signals with normalized log2-transformed transcriptome data. Columns “miRTarbase” and “TargetScan” indicate whether the respective the mRNA has previously been reported as miR-10a or miR-10b target (TRUE or FALSE).

**Supplementary Figures:**

**Supplementary Figure S1: miR-10b target identification and selection.** (A) Heat map of significantly deregulated (≥ 2-fold; p ≤ 0.05) miRNAs. Normalized (RMA), batch corrected (SVA) and z-transformed log2 microarray signals were row-wise clustered by single linkage euclidean distance. (B) KEGG pathway (pwy) enrichment analysis of reported (miRTarbase v6.1) putative targets for ≥ 2-fold, significantly (p-value ≤ 0.05, FDR ≤ 0.2) dysregulated miRNAs (grey dot: RDEB-cSCC vs. RDEB-KC, black dot: HC-cSCC vs HC-KC). (C,D) TaqMan qPCR on primary mir-10a and -10b transcripts, respectively, confirmed differential regulation of miRNA-expression (n = 3). (E) TaqMan qPCR (n = 3) on mature miR-10a-5p indicates mild up-regulation in RDEB-cSCC over RDEB-KC. (F) Both, miR-10a and miR-10b were found to be upregulated in microarray analysis. (G) NGS of AGO2-bound miRNAs isolated from immortalized cell lines, comparing DESeq2 normalized read counts of RDEB-cSCC to immortalized RDEB-KC, confirmed TaqMan results of consistent upregulation of miR-10b. Error bars represent SEM. Statistical tests: t-test (A,C,D,E) and (B) gene set expression analysis (GSEA) test, *p ≤ 0.05 was considered as significant.

**
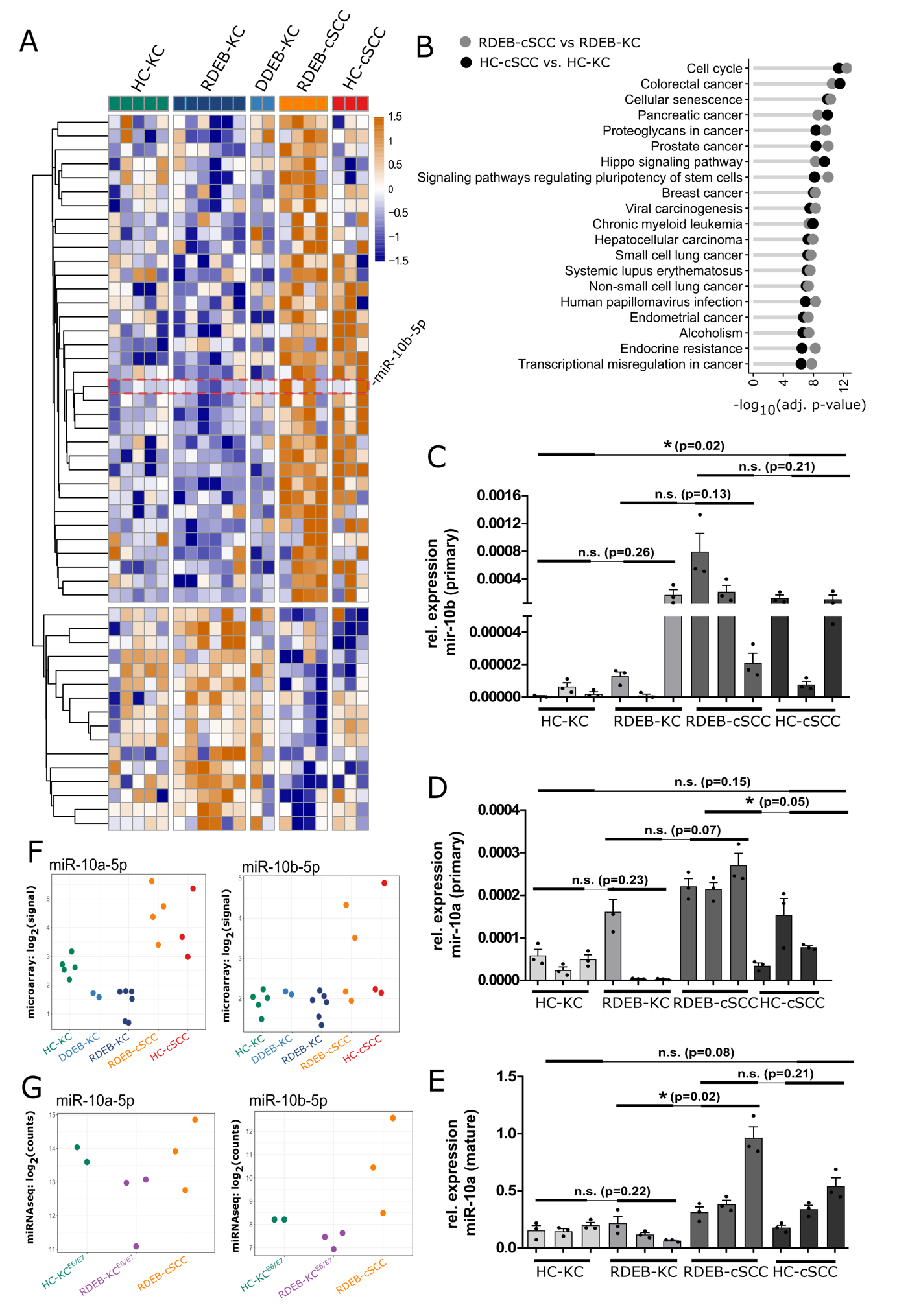
**

**Supplementary Figure S2: Controls for miRNA probe specificity in *in situ* hybridization on FFPE tissue sections.** (A) H&E staining confirms malignant skin transformation, as opposed to HC-skin. (B) Specificity of miR-10b-5p signal (green) was assessed in tumor tissue by comparison of scrambled negative control probe and 2^nd^ step control (no probe) under the same hybridization conditions. Sample processing and hybridization conditions were tested with a positive control probe against *U6* in HC-skin. DAPI was used for nuclei and panKRT (red) for basal KC/SCC staining (C). (D) CD31 and CD45 expression in tissue sections. Immunofluorescence microscopy on HC- and RDEB-SCC tissue sections shows the presence of both, endothelial cells (CD31^+^) and lymphocytes (CD45^+^) to different degrees. (E) In immunofluorescence microscopy, TWIST1 was found to be expressed in RDEB-cSCC tumor sections, but not in HC-skin. (F) Western blot analysis. TWIST1 is highly expressed in RDEB-cSCC cell cultures, as compared to HC-KCs and RDEB-KCs. Alpha-actinin (ACTA) was used as loading control.

**
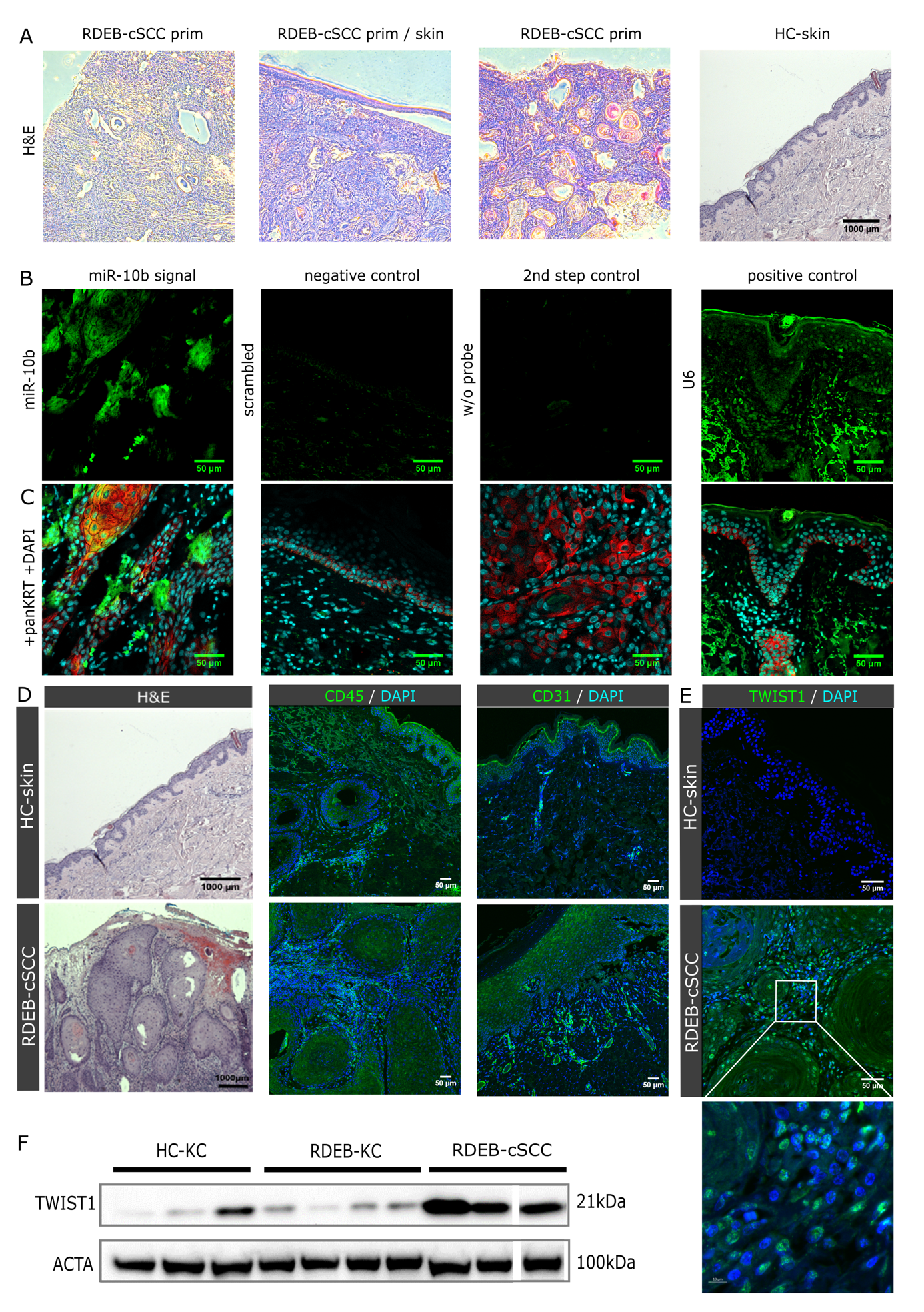
**

**Supplementary Figure S3: miRNA probe specificity for *in situ* hybridization on cultured cells and single cell resolution image-based data analysis workflow.** (A,B) Images were converted to grey scale using the CellProfiler software and the miR-10b specific signal (green) was used for segmentation of cytoplasm (cellular mask) and DAPI (blue) for nuclei (nuclear mask). (C,D) miR-10b specific signals were cumulated within cellular masks for each single cell and reported as “integrated intensity” after subtraction of cumulated nuclear signal, which was most likely derived from unspecific DNA-binding. HC-KC: SKC013. Scale bar = 50 µm.


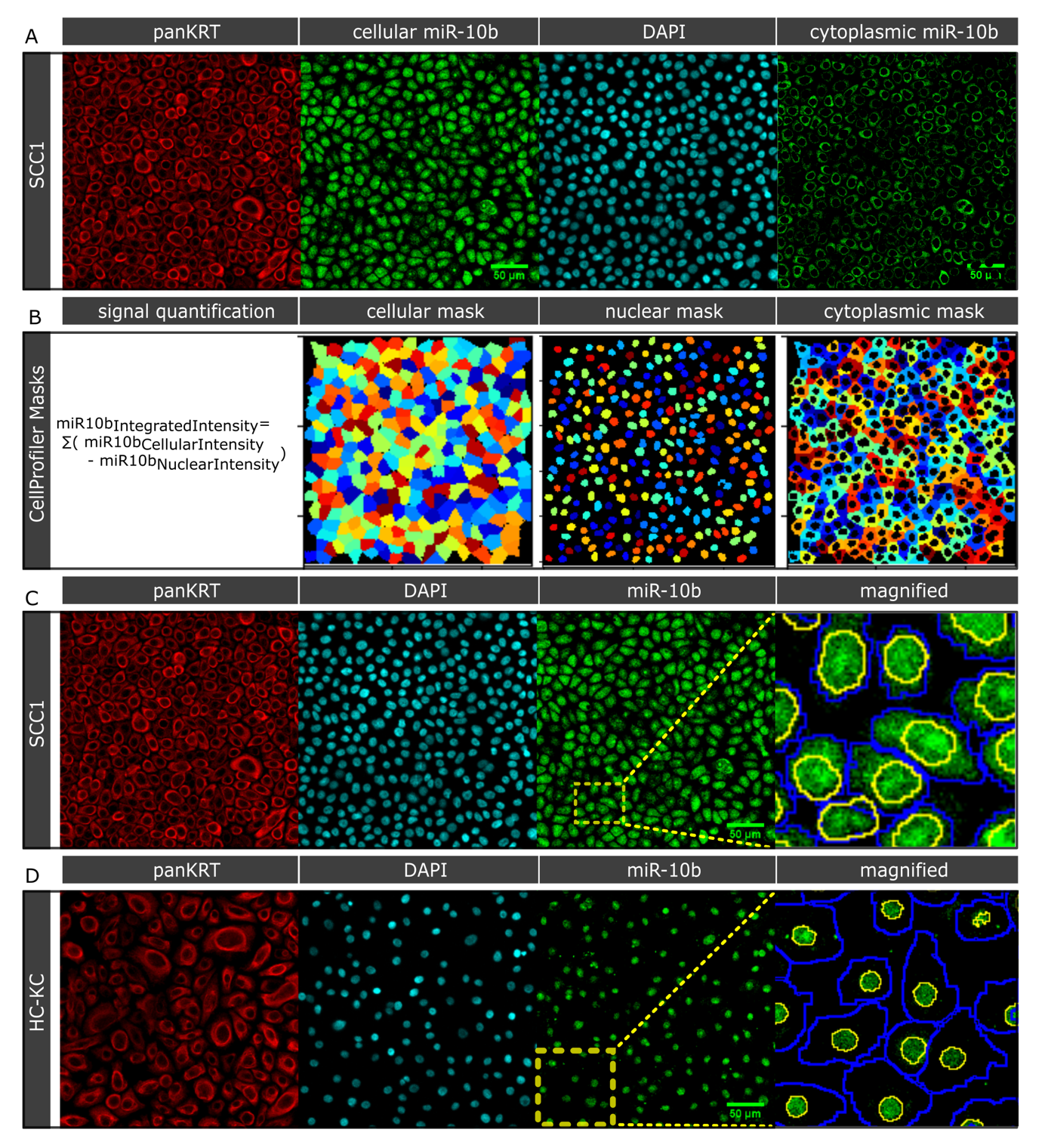


**Supplementary Figure S4: miRNA probe specificity for *in situ* hybridization on cultured cells.** Panel (A) demonstrates miR-10b-5p signal (FITC) in RDEB-cSCC cells. DAPI was used for nuclei and panKRT (red) for cytoplasmic basal KC/SCC staining. (B) Absence of signal when applying a scrambled negative control probe at same hybridization conditions as used for miR-10b-5p probe is shown. In addition a second step control, without any probe was performed to verify non-specific staining of anti-DIG Fab-FITC (C). (D) A probe for *U6* (green) was used as positive control. Scale bar = 50 µm.

**
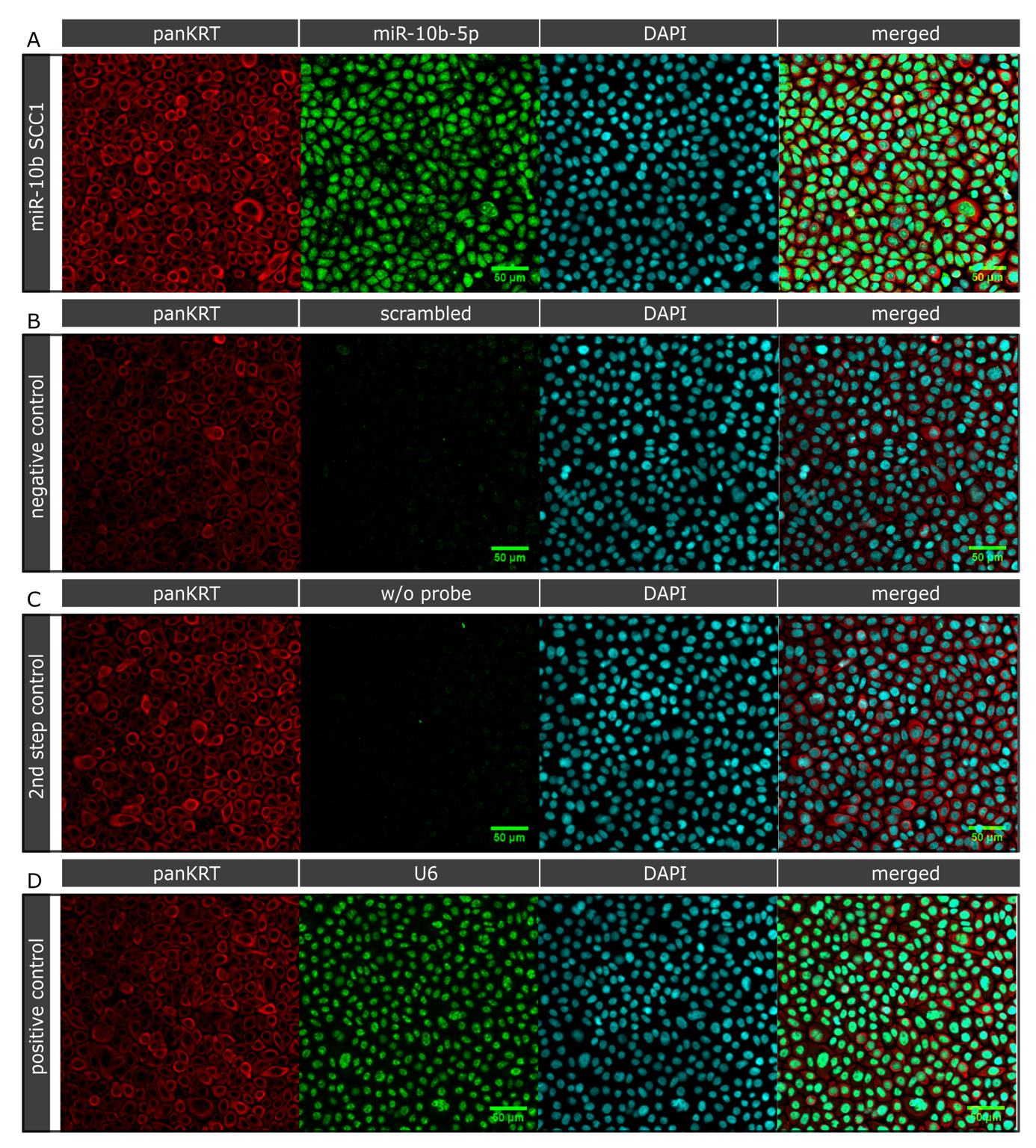
**

**Supplementary Figure S5: Generation of experimental cell lines.** (A) Two different non-malignant KC lines were retrovirally transduced with a *MIR10B* expression cassette under the control of an *U6* promoter. Expression levels of mature miR-10b were assessed by TaqMan qPCR (n = 3 independent repeats; data represented by mean ± SEM, HC-KC: primary 1090KC; RDEB-KC^E6/E7^: immortalized RDEB-57KC). (B) sqRT-PCR confirmed reduced levels of *DIAPH2* in miR-10b overexpressing keratinocytes (n = 3; error bars represent SEM). (C) Upon transfection of 1090KC^E6/E7^ with *DIAPH2* targeted CRISPR/Cas9, single clones were analyzed for successful knock-out of *DIAPH2*. Two clones harboring a homozygous deletion of one nucleotide within exon 1 were used for further experiments. (D) Western blot analysis confirmed knock-out of *DIAPH2* expression upon CRISPR-mediated disruption. (E,F) HC-KC*^DIAPH2-/-^* cells showed a delayed gap closure, similar to miR-10b overexpressing cells (* p < 0.05; n ≥ 8; t-test; error bars represent SEM). The percentage (%) of gap closure compared to the starting area at 0 hrs is given at 3, 6 (red line), 9 and 12 hrs. (G,H) While HC-KC (1090KC) barely formed stable aggregates *in vitro*, knock-out of *DIAPH2* conferred HC-KC the capacity to form stable aggregates. Size distribution of aggregates is similar to that of RDEB-cSCC cells. Representative images of aggregates in parental HC-KC compared to HC-KC*^DIAPH2-/-^* are shown. (I) Viability of cells in aggregates was confirmed via live (calcein-AM) – dead (ethidium) staining after 96 hrs. (J,K) CRISPR/Cas9 knockout of *MIR10B* in RDEB-SCC1 was confirmed by Sanger sequencing and TaqMan qPCR on bulk population, as well as two clones gained by one round of serial dilution (* p < 0.05; n = 3 ind. repeats; unpaired t-test; data represented by mean ± SEM).


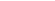


**
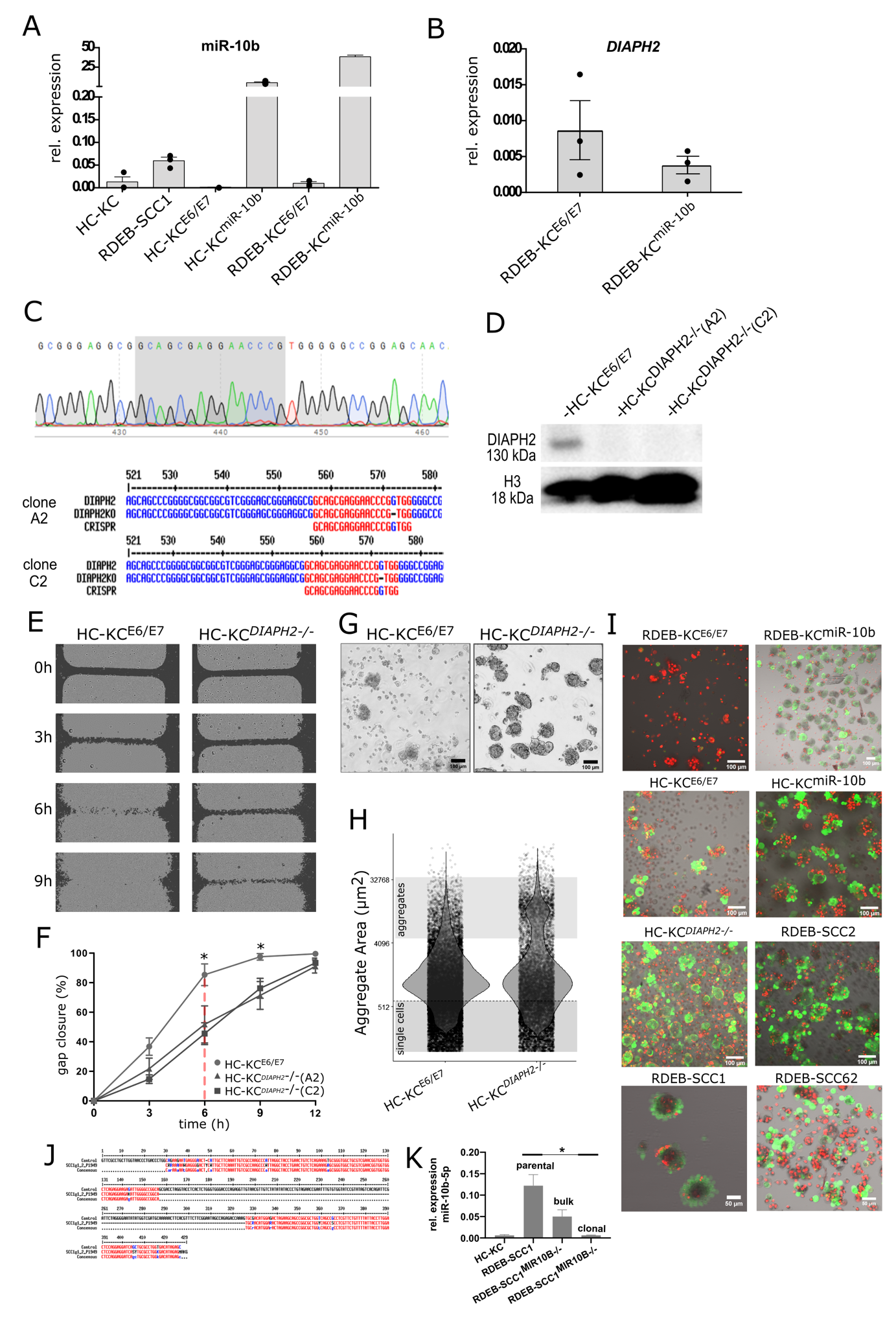
**

**Supplementary Figure S6: Characterization of experimental cell lines.** (A) Generation of cell lines and morphologic characteristics. Experimental cells lines had different sizes, as evaluated by measuring the median cell surface area. For each cell type ≥ 10 cells were analyzed. Those cells stably expressing miR-10b were significantly smaller in size than their parental cells. Also upon *DIAPH2* knock-out, cells became slightly smaller. Analysis of CD44 and CD24: (B) RDEB-KC^miR-10b^ and respective parental cells were analyzed by flow cytometry for surface proteins CD44 and CD24. In the presence of miR-10b, the ratio of CD44^high^/CD24^-/low^ increased from 2.8 % to 11.4 %. Both cell lines expressed similar levels of CD44. (C) RDEB-cSCC cell lines showed overall high expression levels of CD44 and different subpopulations of CD24 expressing cells.

**
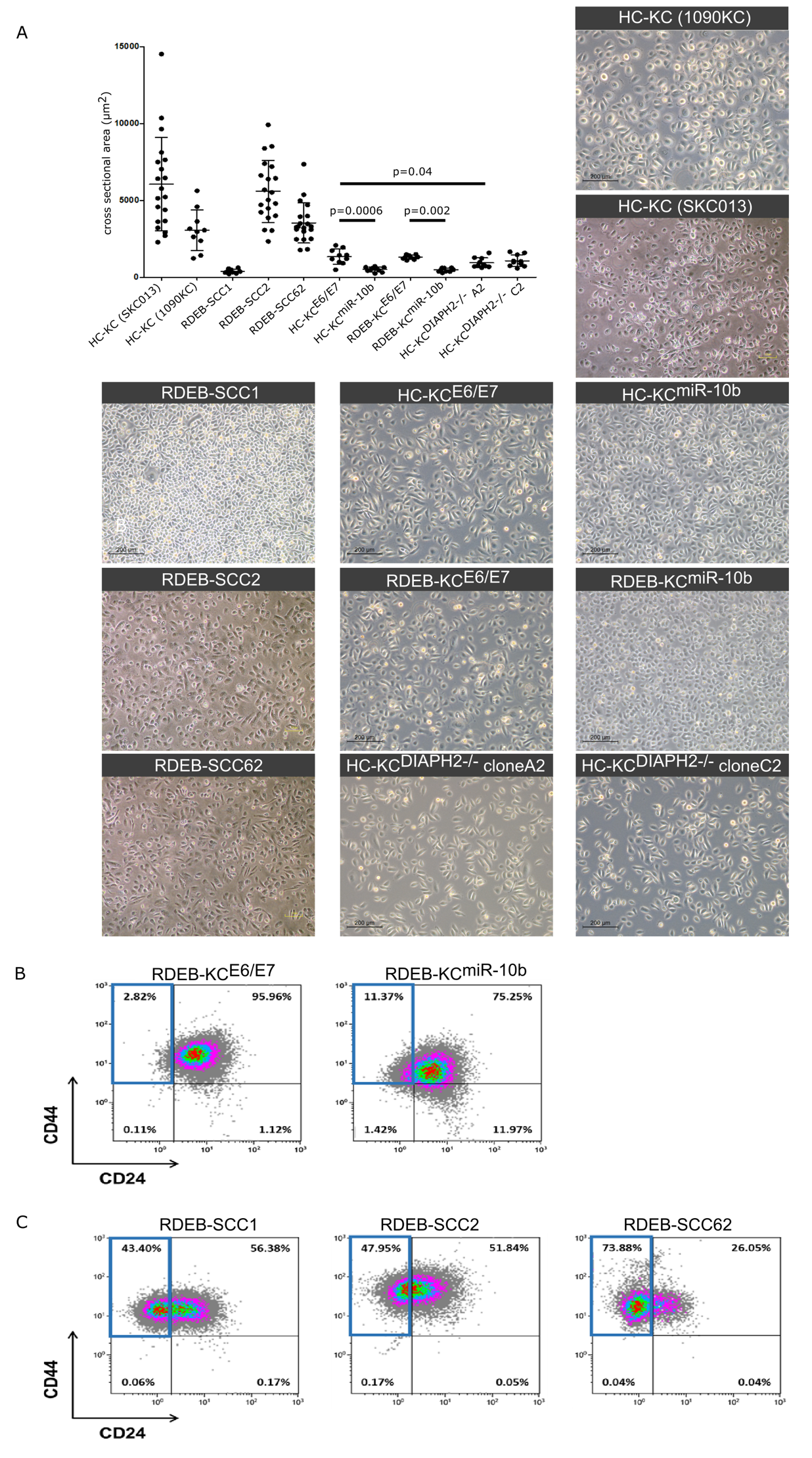
Supplementary Figure S7: Validation of miR-10b targets HOXD10 and DIAPH2.** (A) Scientific literature (1,000 Pubmed-listed articles) was screened for terms “cancer” and “miR-10b” on which named entity recognition (NER) was performed to extract biologically relevant terms (geniatagger). A generated word cloud highlights prominent terms, among others, HOXD10. (B) Validation of *HOXD10* expression by sqRT-PCR did not reveal a downregulation of *HOXD10* mRNA in RDEB-SCC and cSCC lines compared to controls (n = 3; error bars represent SEM; t-test; dashed bars: normalized to GAPDH, plain bars: normalized to TUBA). (C) However, at the protein level HOXD10 was downregulated as shown by immunofluorescence microscopy on cells (HOXD10: red) and (D) on tissue sections of RDEB-skin versus RDEB-cSCC, and HC-skin versus HC-cSCC (HOXD10: green, DAPI:blue was used for nuclear staining). (E) Mean microarray log2 expression signals of miR-10a and miR-10b correlate well (r = 0.77; Pearson) with ΔCq values of miR-10b TaqMan qPCR. Normalized (RMA) log2-transformed microarray expression signals of ≥ 2-fold deregulated genes (RDEB-cSCC vs. RDEB-KC) of the transciptome panel for miR-10 targets in either miRTarbase or Targetscan, were correlated with mean of normalized (RMA) log-2 transformed miR-10 sample-matched microarray expression signals. Among those, DIAPH2 showed a significant (p < 0.01) correlation (r = - 0.57; Pearson). (F) A dual luciferase assay confirmed the direct interaction of a miR-10b mimic with the *HOXD10* 3’UTR and (G) the *DIAPH2* 3’UTR, both cloned downstream the luciferase gene. Luciferase signals decreased on average by 37% when co-transfected with miR-10b mimic for *HOXD10,* and 50% for *DIAPH2*, respectively, compared to a scrambled (scr) control (n = 6; error bars represent SEM; t-test). (H) Upon transfection of HC-KCs with 50 mM miR-10b mimic, DIAPH2 protein levels decreased compared to cells transfected with scr control in dosimetric Western blot analysis (n = 5; normalized to annexin 1; error bars represent SEM; t-test). (I) Validation of *DIAPH2* expression by sqRT-PCR showed downregulation of *DIAPH2* mRNA in RDEB-cSCC compared to HC-KC (n = 3; error bars represent SEM; t-test; dashed bars: normalized to GAPDH, plain bars: normalized to TUBA).

**
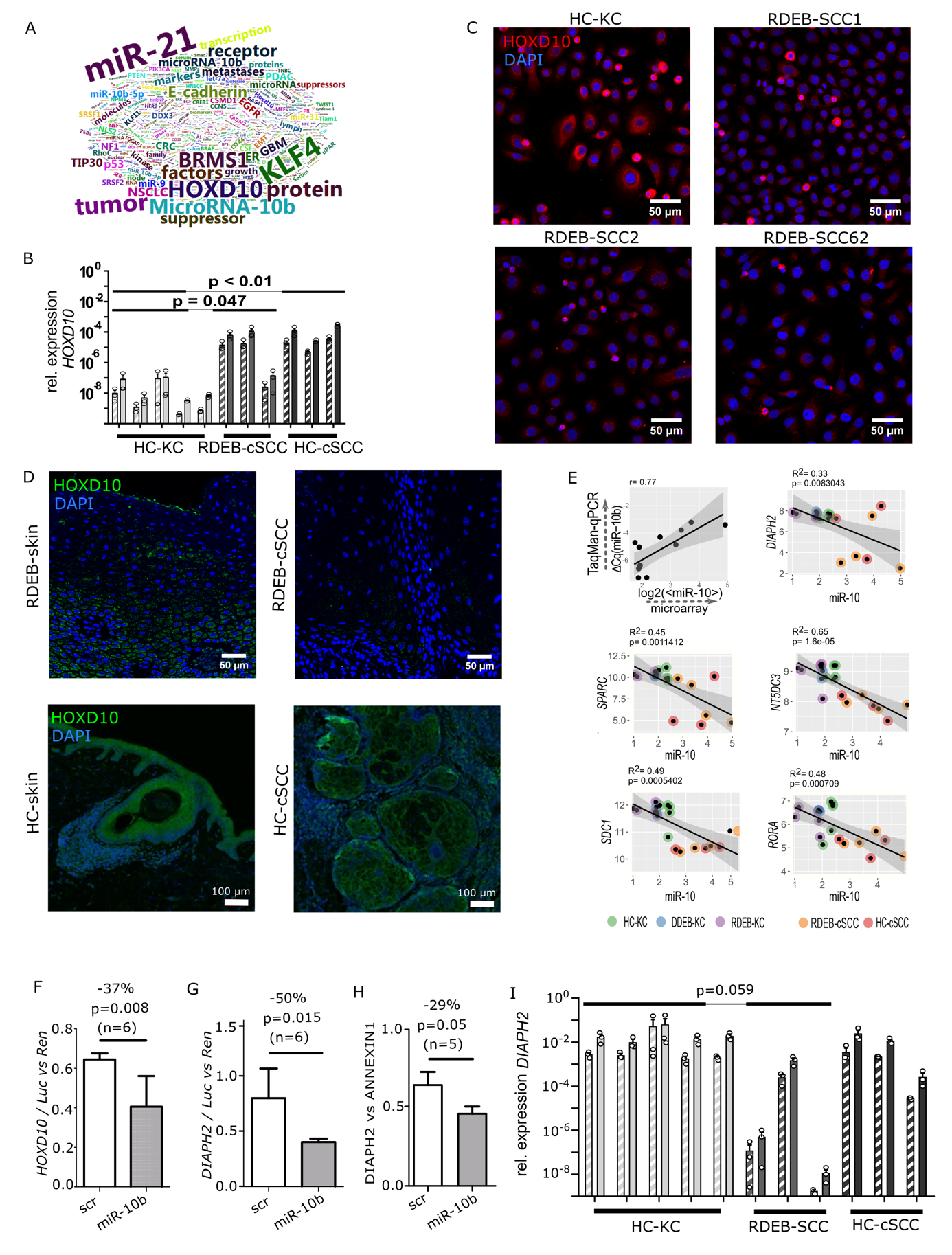
**

**Supplementary Figure S8: Survival analysis and target evaluation.** (A) Survival analysis of miR-10 targets in TCGA HNSCC RNAseq datasets. Stratification of patients based on miR-10 target expression levels results in *DIAPH2* as most significant impact factor on survival (Low: n = 64, high: n = 22, log-rank test). (B) miR-10b targets *DIAPH2*, but not *DIAPH1 and 3,* shows lower expression in RDEB-cSCC (n = 8) compared to RDEB-skin (n = 10; Wilcox test) tissue. (C) As expected, *HOXD10* expression is not reduced at the mRNA level, as it is regulated by translational inhibition. Normalized RNA-seq data generated by Cho *et al* (20), data was retrieved from GEO repository (GSE111582).


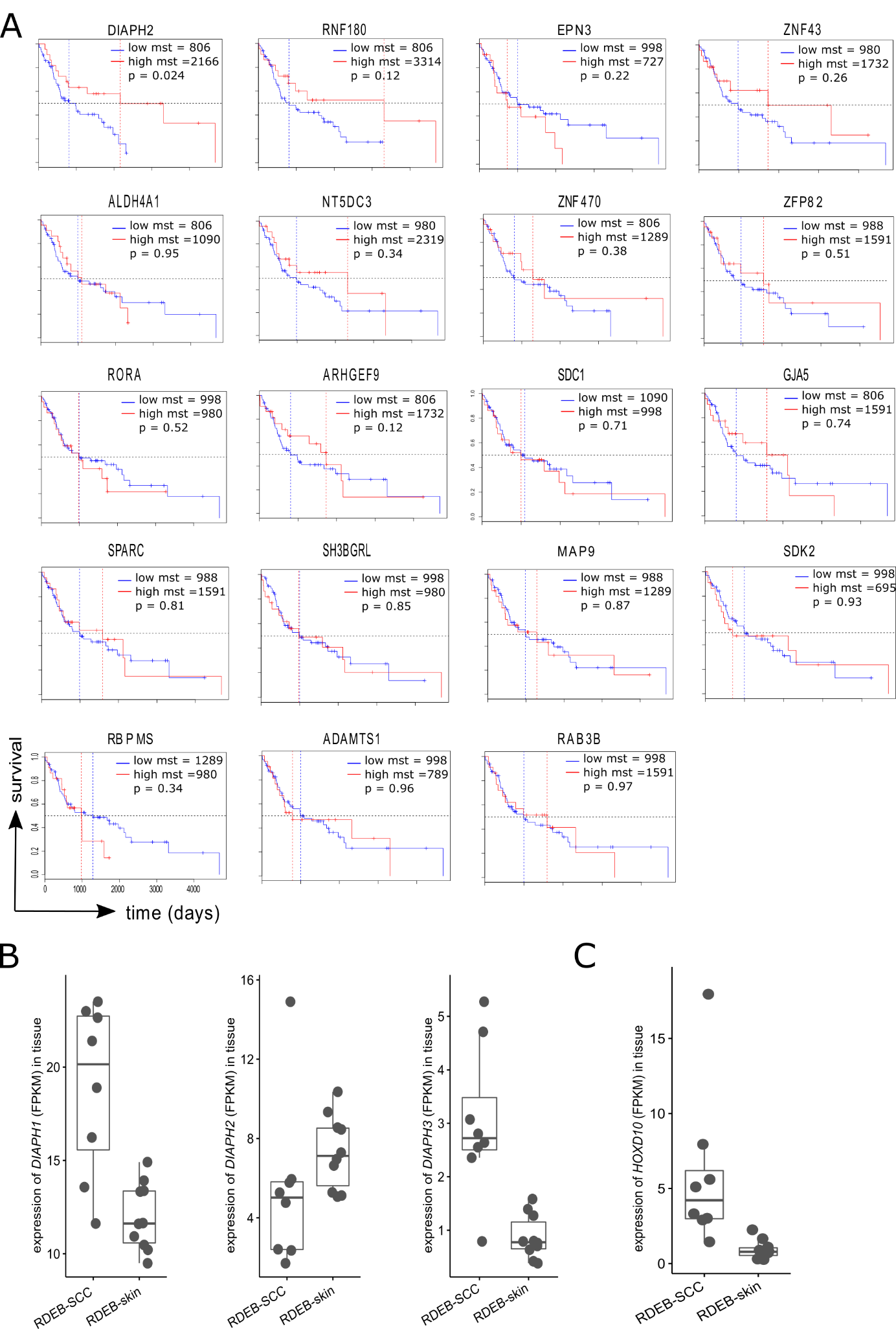


**Supplementary references:**

1. Giard DJ, Aaronson SA, Todaro GJ, Arnstein P, Kersey JH, Dosik H, et al. In vitro cultivation of human tumors: establishment of cell lines derived from a series of solid tumors. J Natl Cancer Inst. 1973;51(5):1417–23.

2. Lu Z, Jiang G, Blume-Jensen P, Hunter T. Epidermal growth factor-induced tumor cell invasion and metastasis initiated by dephosphorylation and downregulation of focal adhesion kinase. Mol Cell Biol. 2001;21(12):4016–31.

3. Rheinwald JG, Beckett MA. Tumorigenic keratinocyte lines requiring anchorage and fibroblast support cultured from human squamous cell carcinomas. Cancer Res. 1981;41(5):1657–63.

4. Goldie SJ, Mulder KW, Tan DW, Lyons SK, Sims AH, Watt FM. FRMD4A upregulation in human squamous cell carcinoma promotes tumor growth and metastasis and is associated with poor prognosis. Cancer Res. 2012;72(13):3424–36.

5. Kocher T, Peking P, Klausegger A, Murauer EM, Hofbauer JP, Wally V, et al. Cut and Paste: Efficient Homology-Directed Repair of a Dominant Negative KRT14 Mutation via CRISPR/Cas9 Nickases. Mol Ther. 2017;25(11):2585–98.

6. Sun Y, Woess K, Kienzl M, Leb-Reichl VM, Feinle A, Wimmer M, et al. Extracellular Vesicles as Biomarkers for the Detection of a Tumor Marker Gene in Epidermolysis Bullosa-Associated Squamous Cell Carcinoma. J Invest Dermatol. 2018;138(5):1197–200.

7. Pourreyron C, Cox G, Mao X, Volz A, Baksh N, Wong T, et al. Patients with recessive dystrophic epidermolysis bullosa develop squamous-cell carcinoma regardless of type VII collagen expression. J Invest Dermatol. 2007;127(10):2438–44.

8. Genesio R, Mormile A, Licenziati MR, De Brasi D, Leone G, Balzano S, et al. Short stature and primary ovarian insufficiency possibly due to chromosomal position effect in a balanced X;1 translocation. Mol Cytogenet. 2015;8:50.

9. Li Y, Liu D, Lopez-Paz C, Olson BJ, Umen JG. A new class of cyclin dependent kinase in Chlamydomonas is required for coupling cell size to cell division. Elife. 2016;5:e10767.

10. Li X, Zheng L, Xia Q, Liu L, Mao M, Zhou H, et al. A novel cell-penetrating peptide protects against neuron apoptosis after cerebral ischemia by inhibiting the nuclear translocation of annexin A1. Cell Death Differ. 2019;26(2):260–75.

11. Shrivastava A, Prasad A, Kuzontkoski PM, Yu J, Groopman JE. Slit2N Inhibits Transmission of HIV-1 from Dendritic Cells to T-cells by Modulating Novel Cytoskeletal Elements. Sci Rep. 2015;5:16833.

12. Eadon MT, Hause RJ, Stark AL, Cheng YH, Wheeler HE, Burgess KS, et al. Genetic Variants Contributing to Colistin Cytotoxicity: Identification of TGIF1 and HOXD10 Using a Population Genomics Approach. Int J Mol Sci. 2017;18(3).

13. Stebbins MJ, Wilson HK, Canfield SG, Qian T, Palecek SP, Shusta E V. Differentiation and characterization of human pluripotent stem cell-derived brain microvascular endothelial cells. Methods. 2016;101:93–102.

14. Fan JR, Lee HT, Lee W, Lin CH, Hsu CY, Hsieh CH, et al. Potential role of CBX7 in regulating pluripotency of adult human pluripotent-like olfactory stem cells in stroke model. Cell Death Dis. 2018;9(5):502.

15. Pettersson AT, Laurencikiene J, Mejhert N, Naslund E, Bouloumie A, Dahlman I, et al. A possible inflammatory role of twist1 in human white adipocytes. Diabetes. 2010;59(3):564–71.

16. Nagle RB, Lucas DO, McDaniel KM, Clark VA, Schmalzel GM. Paget’s cells. New evidence linking mammary and extramammary Paget cells to a common cell phenotype. Am J Clin Pathol. 1985;83(4):431–8.

17. Fusco DN, Pratt H, Kandilas S, Cheon SS, Lin W, Cronkite DA, et al. HELZ2 Is an IFN Effector Mediating Suppression of Dengue Virus. Front Microbiol. 2017;8:240.

18. Moreno N, Gonzalez A. Pattern of Neurogenesis and Identification of Neuronal Progenitor Subtypes during Pallial Development in Xenopus laevis. Front Neuroanat. 2017;11:24.

19. Schober AL, Gagarkin DA, Chen Y, Gao G, Jacobson L, Mongin AA. Recombinant Adeno-Associated Virus Serotype 6 (rAAV6) Potently and Preferentially Transduces Rat Astrocytes In vitro and In vivo. Front Cell Neurosci. 2016;10:262.

20. Cho RJ, Alexandrov LB, den Breems NY, Atanasova VS, Farshchian M, Purdom E, et al. APOBEC mutation drives early-onset squamous cell carcinomas in recessive dystrophic epidermolysis bullosa. Sci Transl Med. 2018;10(455).
